# Magnetically tunable singlet-triplet spin qubit in a four-electron InGaAs coupled quantum dot

**DOI:** 10.1038/srep03121

**Published:** 2013-11-01

**Authors:** K. M. Weiss, J. Miguel-Sanchez, J. M. Elzerman

**Affiliations:** 1 Institute for Quantum Electronics, ETH Zurich, CH-8093 Zurich, Switzerland; 2 London Centre for Nanotechnology and Department of Electronic & Electrical Engineering, University College London, London WC1H 0AH, UK

## Abstract

A pair of self-assembled InGaAs quantum dots filled with two electrons can act as a singlet-triplet spin qubit that is robust against nuclear spin fluctuations as well as charge noise. This results in a *T*_2_* coherence time two orders of magnitude longer than that of a single electron, provided the qubit is operated at a particular “sweet spot” in gate voltage. However, at this fixed operating point the ground-state splitting can no longer be tuned into resonance with e.g. another qubit, limiting the options for coupling multiple qubits. Here, we propose using a *four-electron* coupled quantum dot to implement a singlet-triplet qubit that features a magnetically tunable level splitting. As a first step towards full experimental realization of this qubit design, we use optical spectroscopy to demonstrate the tunability of the four-electron singlet-triplet splitting in a moderate magnetic field.

A single electron spin confined in a self-assembled InGaAs quantum dot (QD) can be conveniently initialised, manipulated and read out using laser pulses^1^. However, it interacts strongly with the bath of fluctuating nuclear spins that is inevitably present in all III-V semiconductors, limiting its *T*_2_* coherence time to just a few nanoseconds^2^,^3^,^4^. To overcome this issue, a promising strategy is to encode the qubit in “atomic-clock states” that are insensitive (to first order) to both nuclear spins and charge fluctuations^5^. A coupled quantum dot (CQD) filled with two electrons features spin singlet and triplet ground states^6^,^7^,^8^ that can be used for this purpose. This results in an increase of *T*_2_* by at least two orders of magnitude^8^, provided the system is operated at a particular “sweet spot” in gate voltage where it is immune to charge noise^9^,^10^.

However, because the two-electron qubit states are insensitive to magnetic field and the operating point is fixed at the sweet spot, there are no control mechanisms available to tune the qubit states *in situ*. The singlet-triplet splitting for a particular two-electron CQD at the sweet spot is thus fully determined by microscopic parameters (such as the exact size of the QDs and their separation), which vary substantially from dot to dot. This lack of control is problematic for quantum information processing tasks, which would benefit from the ability to bring qubits into resonance with each other or with a shared quantum bus^11^. In addition, qubit tunability would ease experimental demands in coupling schemes relying on Raman transitions in an optical cavity^12^, or on dipolar interactions with a ferromagnet^13^.

Here, we propose a four-electron version of the two-electron singlet-triplet qubit that allows the ground-state splitting to be efficiently tuned with a moderate magnetic field. To verify the feasibility of the proposed qubit design, we experimentally investigate a self-assembled InGaAs CQD filled with four electrons. We use high-resolution magneto-optical spectroscopy to demonstrate the tunability of the four-electron singlet-triplet splitting. In addition, we establish that the qubit states form a lambda system with a shared optically excited state, which is a crucial feature enabling fast optical manipulation^6^,^14^,^15^,^16^,^17^,^18^. Finally, we identify an unusually fast spin relaxation channel that compromises the qubit's coherence time, and we suggest a straightforward way to circumvent it.

## Results

### Four-electron singlet-triplet qubit states

**Figure 1:**
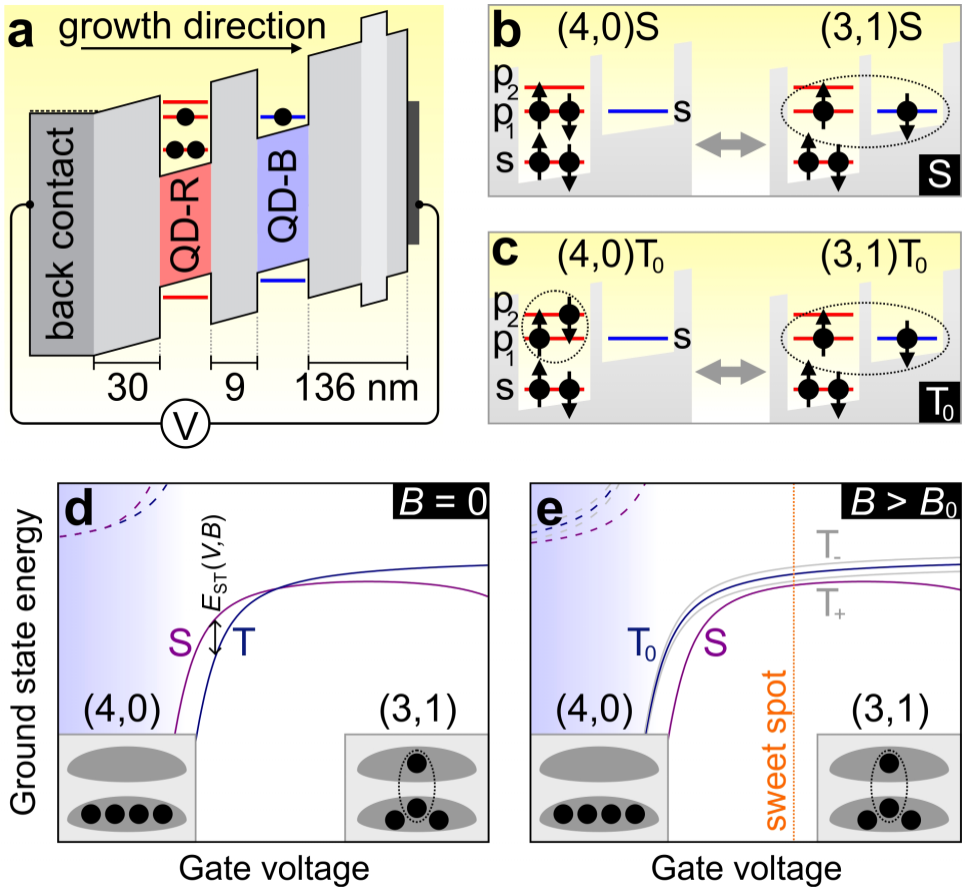
Four-electron singlet-triplet qubit. (a) Schematic energy diagram of the device in the (3,1) charge configuration, i.e. with three electrons (indicated by black circles) in the lower red-detuned dot (QD-R) and one in the upper blue-detuned dot (QD-B). (b,c) Schematic depiction of the four-electron qubit states. The spin singlet *S* is a bonding superposition of mainly states (4,0)*S* and (3,1)*S*. Similarly, the spin triplet *T*_0_ is a bonding superposition of (4,0)*T*_0_ and (3,1)*T*_0_. These states involve the s-orbital of both dots as well as the *p*_1_ and *p*_2_ orbital of QD-R. A more detailed description of all the components of the spin superpositions is given in supplementary Fig. S2. The dashed circles denote the antisymmetric spin superposition (↑↓ − ↑↓)/√2 in the case of the S state, and the symmetric spin superposition (↑↓ + ↑↓)/√2 in the case of *T*_0_. (d) Energies of the four-electron CQD states versus *V* for *B* = 0. The three degenerate triplet states (labelled *T*) are split off from *S* by a voltage-dependent exchange splitting *E*_ST_ = *E*_S_ − *E*_T0_. Dashed lines indicate anti-bonding singlet (purple) and triplet (blue) states not used in the experiment. Insets: the configuration of electrons (black circles) residing in the quantum dots (grey). (e) Energies of the CQD states in a magnetic field *B* > *B*_0_ along the growth direction, which shifts (4,0)*S* below (4,0)*T*_0_. This leads to the appearance of a sweet spot in *V* (dotted orange line), where d*E*_ST_/d*V* = 0. The triplet states with both high-energy electrons pointing up (*T*_+_) or down (*T*_−_) along *B* are split off from *T* by the Zeeman energy, and are indicated in grey.

The tunable qubit design we propose is based on two coupled quantum dots containing four electrons (Fig. 1a). Two low-energy electrons reside permanently in the s-orbital of the red-detuned dot (QD-R), forming a spin singlet. The spin character of the four-electron states is determined by the two high-energy electrons, which are distributed over the p-orbitals (*p*_1_ and *p*_2_) in QD-R and the s-orbital in the blue-detuned dot (QD-B). To ensure immunity against nuclear spins, the qubit is encoded in the two lowest-energy states with zero total spin-projection along the sample growth direction *z*, i.e. the spin singlet *S* and the spin triplet *T*_0_. A schematic depiction of *S* and *T*_0_ is shown in Figs. 1b and 1c, and a more detailed description is given in supplementary Fig. S2. We will mostly ignore the other two spin triplet states, which have both spins pointing up (*T*_+_) or down (*T*_−_) along *z*.

The magnetic tunability of the qubit is due to the orbital angular momentum of *p*_1_ and *p*_2_, whose energies shift in opposite directions when we apply a magnetic field *B* along *z* (Ref. 19). Around zero field, where the p-state splitting δε_p_ is small, it is energetically favourable for one electron to occupy *p*_2_ and form a spin-triplet with the *p*_1_-electron, lowering their combined energy by the exchange term *K* ~ 1 meV (Ref. 20). Upon increasing the field, δε_p_ grows until it equals *K* at *B*_0_ ~ 1 T (depending on the electronic effective mass in the CQD and the asymmetry of its confining potential). For *B* > *B*_0_ the ground state is a spin singlet with both high-energy electrons occupying *p*_1_.

This magnetic-field induced singlet-triplet transition is well-known from single QDs containing four electrons^19^,^20^. In our coupled QD system, inter-dot tunnelling mixes the (4,0) states (where all four electrons are located in QD-R) with the (3,1) states (where one electron has tunnelled to QD-B). Since tunnelling conserves spin, there is an anti-crossing involving the singlet states (4,0)*S* and (3,1)*S*, and a separate one involving the triplets (4,0)*T*_0_ and (3,1)*T*_0_. At zero magnetic field both anti-crossings occur at slightly different values of the gate voltage *V*, due to the energy difference between (4,0)*S* and (4,0)*T*_0_. This results in a *V*-dependent exchange splitting *E*_ST_ between *S* and *T*_0_ (Fig. 1d). Towards larger voltages, *S* is pulled down in energy and actually crosses *T*_0_, due to mixing with the (2,2) singlet state (see also supplementary Fig. S1b). As a result, a sweet spot where d*E*_ST_/d*V* = 0 (such that the qubit splitting is insensitive to first order to charge noise) does not exist anywhere across the gate-voltage range under these conditions. The sweet spot appears only for *B* > *B*_0_, where the spin singlet is the ground state (Fig. 1e). If *B* is increased even further, the sweet spot moves towards larger *V* and the singlet-triplet splitting increases (see supplementary Fig. S3). This *in situ* tunability sets the four-electron singlet-triplet qubit apart from its two-electron counterpart^6^,^8^.

### Lambda system at zero magnetic field

**Figure 2:**
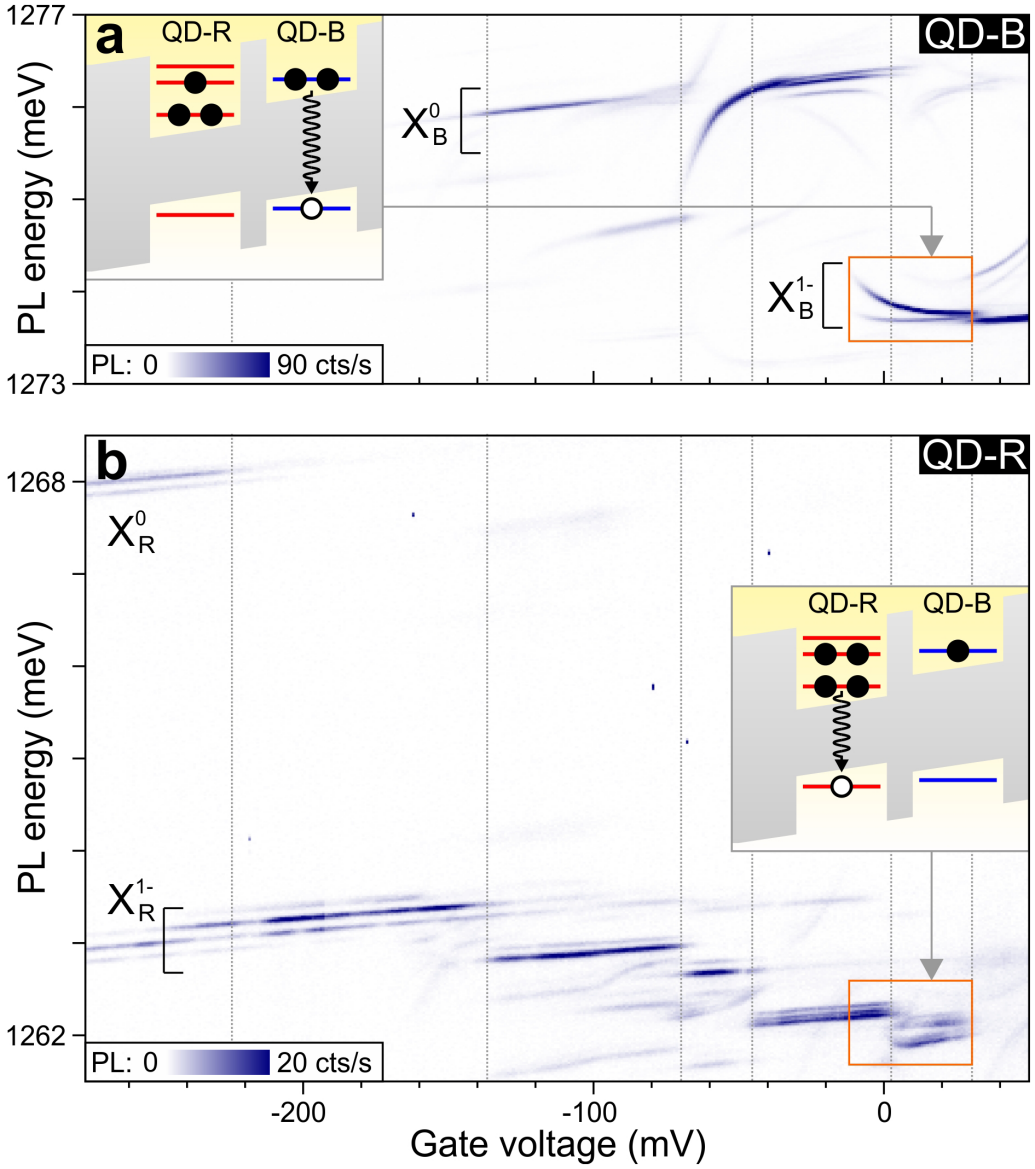
Locating the four-electron regime at *B* = 0 T. (a) PL from QD-B (in colorscale) as a function of *V*. Dotted vertical lines indicate the gate voltages where the charge configuration of the optically excited states changes. *X*_B_^0^ (*X*_B_^1−^) indicates emission from the neutral exciton (negative trion) in QD-B. PL involving the four-electron *S* and *T* ground states (highlighted in the orange box) exhibits a characteristic curvature. The larger signal of the *T* transition is due to the threefold degeneracy of the spin triplets. Inset: schematic energy diagram illustrating *X*_B_^1−^ emission in the (3,1) regime. (b) PL from QD-R (in colorscale), which is weaker than that from QD-B because holes can tunnel from QD-R to QD-B before recombination. *X*_R_^0^ (*X*_R_^1−^) indicates emission from the neutral exciton (negative trion) in QD-R. The significant overlap between the *X*_R_^0^ and *X*_R_^1−^ transition below −220 mV suggests that the tunnelling rate between the s-orbital and the back contact is slower than the radiative recombination rate of ~1 GHz. In contrast, the sharp transitions between plateaus above −140 mV indicate that the tunnelling rate from the p-orbitals to the back contact is larger than ~1 GHz. The multiple “satellite lines” that are especially strong for *X*_R_^1−^ are most likely due to fluctuations in the charge of QD-B, leading to a shift in PL due to charge sensing^33^. Inset: schematic energy diagram illustrating emission in the (3,1) regime, which involves the s-orbital in QD-R, and therefore does not reflect the anti-crossings involving the p-orbitals.

To implement the four-electron qubit experimentally, we select a pair of tunnel-coupled self-assembled InGaAs QDs^21^,^22^ where the lower dot is ~6 nm red-detuned from the upper one. This unusual configuration ensures that QD-R is charged with three electrons before the first electron enters QD-B. From the voltage-dependent photoluminescence (PL) at *B* = 0 T, we identify the region in *V* where the CQD contains four electrons. The PL from QD-B clearly reflects the anti-crossings for both singlet and triplet states (highlighted in the orange box in Fig. 2a). The *S* transition can be identified by its ~3 times weaker intensity compared to the transition involving the threefold degenerate *T* states. From the shape of the anti-crossing, we find the inter-dot tunnelling rate between the s-orbital in QD-R and the p-orbitals in QD-B to be ~60 GHz. There are no signs of the anti-crossings in the PL from QD-R (orange box in Fig. 2b), since this involves recombination from its low-lying s-orbital, which does not tunnel-couple to QD-B due to the large energy difference (see the inset to Fig. 2b).

**Figure 3:**
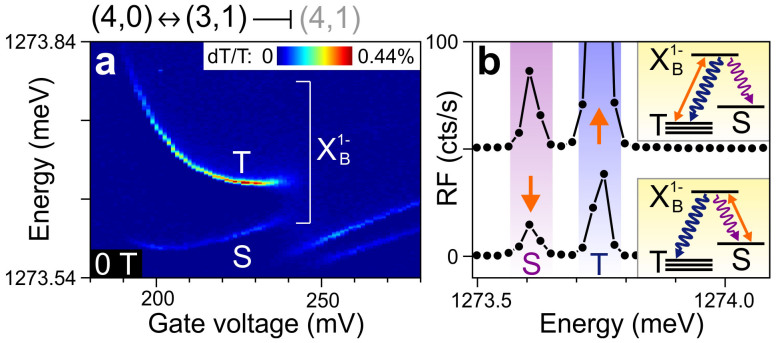
Identifying the four-electron singlet-triplet lambda system at *B* = 0 T. (a) Differential transmission (dT/T) of the singly-charged trion transitions in QD-B (*X*_B_^1−^) versus *V*. In the absence of a magnetic field, the three degenerate triplet states *T*_0_, *T*_+_ and *T*_−_ are strongly mixed by hyperfine effects and appear as a single transition (labelled *T*). The (3,1) charge regime extends to ~240 mV; at larger *V* a fifth electron enters the CQD to form the (4,1) charge configuration which does not have spin singlet or triplet character. (b) Resonance fluorescence at *V* = 200 mV. The *T* transition (upper trace) or the *S* transition (lower trace) is driven resonantly, and the resulting resonance fluorescence is detected by a grating spectrometer. The upper trace is offset vertically for clarity. In addition to a peak at the energy of the driving laser (orange arrows), a second peak is seen at the non-driven transition, demonstrating that the *S* and *T* ground states indeed form a lambda system with the shared optically excited state labelled *X*_B_^1−^. (The right peak in the upper trace is strongly enhanced due to imperfect suppression of the driving laser.) Insets: schematic diagrams of laser driving and photon emission in the lambda system formed by *S*, *T* and *X*_B_^1−^.

To study the four-electron qubit states in more detail, we use single-laser differential transmission (dT). This technique is not well suited to probe the p-orbitals in QD-R directly, since the optical selection rules dictate that generating a p-state electron leaves behind a p-state hole, which will relax very quickly leading to a very broad dT linewidth^23^. Therefore, we use standard resonant s-to-s excitation^24^ to probe *X*_B_^1−^, the singly negatively charged trion in QD-B. We first map out the *S* and *T* transitions at *B* = 0 T (Fig. 3a). As in the PL measurements, both transitions show a separate anti-crossing. They change abruptly around *V* = 240 mV, signaling that here a fifth electron can enter the CQD, corresponding to the (4,1) charge regime.

Next, we verify that both transitions indeed involve the same optically excited state, so that the qubit can be operated as a lambda system^6^,^8^. We fix the gate voltage around 200 mV and detect the resonance fluorescence^25^,^26^ when driving either the *T* transition (upper trace in Fig. 3b) or the *S* transition (lower trace in Fig. 3b). In both cases, a peak is seen at the non-driven transition (in addition to the peak indicated by the orange arrows, which occurs at the energy of the driving laser). This is clear evidence that the *S* and *T* ground states indeed share an optically excited state, making the qubit suitable for fast optical manipulation^6^,^14^,^15^,^16^,^17^,^18^.

Upon closer scrutiny, the results in Fig. 3 are very surprising: due to the lambda configuration, a laser close to saturation power and tuned to the *S* (*T*) transition will very quickly (after at most a few nanoseconds) drive the system to the *T* (*S*) state, where it is strongly detuned from the driving field and thus unable to scatter any more laser photons. This spin shelving^27^,^28^ should result in the disappearance of the dT signal away from the edge of the (3,1) regime at 240 mV. However, the dT contrast in Fig. 3a does not vanish throughout the 190–240 mV region, pointing to efficient (~1 GHz) spin-relaxation between the *S* and *T* states that undoes the spin shelving. In previous experiments using a two-electron ST qubit we observed a similar effect, which we could attribute to strong spin-flip cotunnelling with the nearby back contact^7^. We verified that with a 3 T magnetic field applied along the growth direction (i.e. in the Faraday geometry), the present CQD shows normal spin shelving in the (1,0) regime when driving the *X*_R_^1−^ transition, which involves the low-lying s-orbital in QD-R. This indicates that the 30 nm tunnel barrier to the back contact is sufficiently thick to allow good isolation of the s-orbital (i.e. a spin-flip cotunnelling rate below a few MHz). However, the p-orbitals have a larger lateral size^19^ and a ~20 meV higher energy in the QD potential well, making a larger overlap with the electronic states in the back contact possible and leading to faster spin-flip cotunnelling (with a rate of ~1 GHz).

We conclude that a sample with a thicker tunnel barrier to the back contact is required in order to provide good isolation of the p-states. Increasing the tunnel barrier from 30 nm to 50 nm should reduce the cotunnelling rate for the p-orbitals below ~1 MHz, which would be sufficient to ensure it no longer limits the coherence time (which could be as long as ~1 μs). Although such a thick tunnel barrier would also strongly reduce tunnel coupling from the back contact to the s-orbital in QD-R, this will not jeopardize the functionality of the device, since the CQD system can still re-initialize to the correct charge configuration via the p-orbitals.

### Tuning the qubit with magnetic field

**Figure 4:**
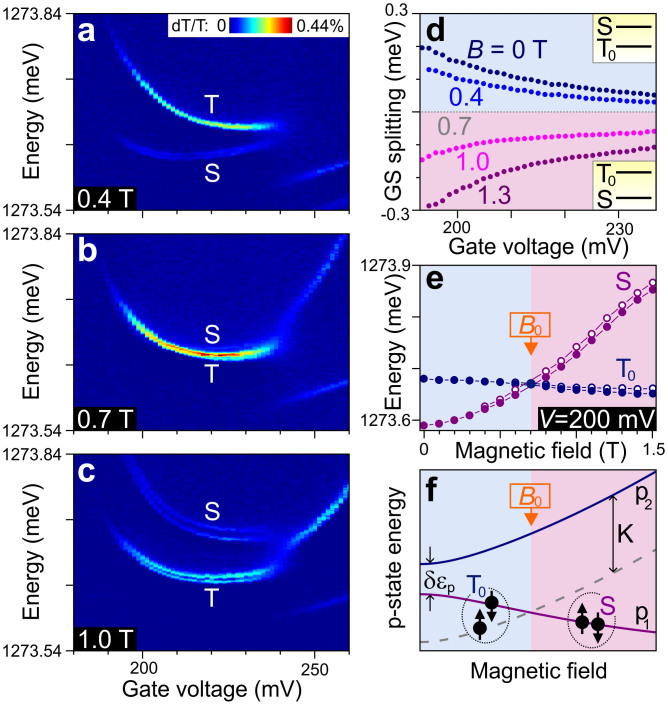
Magnetic tunability of the four-electron singlet-triplet qubit. (a,b,c) Differential transmission (dT/T) of *X*_B_^1−^ versus *V*, for three values of the magnetic field. At 0.4 T, the *T*-manifold forms the ground state (and thus has the higher optical transition energy); at 0.7 T, *S* and *T*_0_ are close to degenerate; and at 1.0 T, the *S* state is lowest in energy. The abrupt change in the optical spectrum at *V* ~ 240 mV signals the start of the (4,1) charge regime. (d) Ground-state splitting (*E*_ST_ = *E*_S_ − *E_T_*_0_) versus *V*, for five values of the magnetic field. The dashed grey horizontal line indicates that the ground-state splitting at *B* = 0.7 T is approximately equal to zero throughout the (3,1) regime. (e) Energy of the *S* and *T*_0_ transitions versus *B* at *V* = 200 mV. The doubling of each transition is due to the (unusually small) Zeeman splitting of the optically excited trion state *X*_B_^1−^. The magnetic field at which *S* and *T*_0_ are approximately degenerate is labelled *B*_0_. (f) Schematic energy diagram of the two p-orbitals (*p*_1_ and *p*_2_) in QD-R versus *B*. The splitting at *B* = 0 results from asymmetry in the confining potential of QD-R. It increases with *B* due to the opposite orbital angular momentum of *p*_1_ and *p*_2_. The dashed grey line follows *p*_2_ with the two-electron exchange energy *K* subtracted. At *B*_0_, the splitting of the p-orbitals δε_p_ equals *K*, so the spin configuration of the ground state switches from spin triplet (for *B* < *B*_0_) to spin singlet (for *B* > *B*_0_).

To demonstrate the magnetic tunability of the qubit states, we map out the optical transitions at various magnetic fields, applied along the growth direction. At 0.4 T (Fig. 4a) the *S* transition has clearly moved closer to the *T*_0_ transition. At 0.7 T (Fig. 4b), the two transitions overlap, indicating that the ground states are practically degenerate throughout the gate voltage range. (Close inspection suggests that the transitions in fact cross around 210 mV, as expected, but the measurement resolution is not sufficient to say this with certainty.) Above 0.7 T (Fig. 4c), both transitions exhibit a clear Zeeman splitting (which is due to the splitting of the optically excited states as *S* and *T*_0_ themselves do not split). The larger contrast of the *T*_0_ transitions, which can be seen for all magnetic fields, is due to the fact that they coincide with transitions involving the other two triplets, *T*_+_ and *T*_−_ (Ref. 8).

From these and similar measurements we construct Fig. 4d, which shows how the splitting between *S* and *T*_0_ changes with *B* across the gate-voltage range. From the figure it is clear that the sweet spot (which is expected for *B* > *B*_0_ = 0.7 T) lies just outside the ~190–240 mV region where (3,1) is the stable charge configuration. Thus, the sweet spot cannot be reached in this particular CQD. At first glance, tuning the magnetic field to *B*_0_ (where *S* and *T*_0_ practically overlap and thus the slope of *E*_ST_ vanishes as well) might seem to be an alternative. However, here the protection of the qubit states against nuclear spins is lost, since *E*_ST_ is comparable to the typical energy of the nuclear spin fluctuations, *E*_n_ ~ 1 μeV.

The shift of the transitions versus *B* is analysed in more detail in Fig. 4e, which focuses on *V* = 200 mV. The *T*_0_-transition remains nearly constant, whereas the *S* transition goes up quickly in energy. This behaviour can be understood from the expected shift of *p*_1_ and *p*_2_ with *B* (Fig. 4f). State *T*_0_ involves both *p*_1_ and *p*_2_, which move in opposite directions with *B*, nearly cancelling each other's shift^19^. On the other hand, *S* only involves *p*_1_, which shifts down strongly with *B*.

The behaviour of the transitions around zero field depends on the symmetry of the dot. If the confining potential of QD-R were perfectly symmetric, the p-splitting δε_p_ would vanish at *B* = 0 and increase linearly with *B*. This would show up in Fig. 4e as a non-zero slope for the S transition around *B* = 0. In our case, asymmetry in QD-R gives rise to a zero-field splitting between *p*_1_ and *p*_2_ (Fig. 4f), corresponding to a vanishing slope around *B* = 0 in Fig. 4e. The exact value of *B*_0_ also depends on the asymmetry; our measured value of *B*_0_ = 0.7 T is actually very close to that reported in Ref. 20. It is important to note that the qubit's insensitivity to nuclear spins is not compromised by the strong *B*-dependence of *E*_ST_, since this is an orbital effect, whereas a nuclear spin polarization leads to a Zeeman-like interaction that is cancelled for both *S* as well as *T*_0_.

## Discussion

In conclusion, we have proposed a four-electron singlet-triplet qubit that allows magnetic control over the ground state splitting. If the singlet-triplet splitting is sufficiently large (*E*_ST_ ≫ *E*_n_, where *E*_n_ ~ 1 μeV is the typical energy corresponding to the statistical fluctuations in nuclear spin-polarization), the qubit should be immune to nuclear spin fluctuations. In addition, when the ground state is the spin singlet (which is the case for sufficiently large magnetic field *B* > *B*_0_), the qubit features a sweet spot where its first-order sensitivity to charge fluctuations^9^,^10^ vanishes. Although these low-frequency fluctuations can have an rms amplitude of just a few μV in high-quality material^9^,^10^, they limit the *T*_2_* time of singlet-triplet qubits away from the sweet spot to ~90 ns (Ref. 10) or even less than a nanosecond (Ref. 6).

Whether or not the four-electron sweet spot lies within the voltage-range where the (3,1) charge configuration is stable depends crucially on the alignment of the single-dot energy levels, in complete analogy to the two-electron case^6^,^7^,^8^. Since we cannot tune the potential energy of each QD separately, we have to carefully select a CQD pair where each dot has the appropriate emission wavelength (to within ~0.2 nm). If the bottom QD wavelength is too red, or the top QD is too blue, then the four-electron sweet spot will be close to the rightmost edge of the (3,1) plateau or even outside it (as was the case in the CQDs we investigated).

In the sample we studied, the p-states suffered from fast (~1 GHz) spin-flip cotunnelling via the back contact, limiting the qubit's coherence time. The cotunnelling rate can be reduced to less than 1 MHz by using a sample with a thicker (~50 nm) tunnel barrier. In that case, the remaining decoherence mechanisms are expected to be similar to those for a two-electron singlet-triplet qubit^2^,^11^, despite the weaker confinement of the p-states and correspondingly larger coupling to the wetting layer^20^. Thus, we expect a similar coherence time for the four-electron qubit, i.e. *T*_2_* > 0.2 μs, possibly extending up to 1 μs (Ref. 8).

In practice, each CQD pair will be slightly different due to the natural spread in parameters such as the confinement potential asymmetry. Therefore, each CQD will require a different combination of *V* and *B* to reach a particular singlet-triplet splitting. Tuning separate CQD pairs into resonance will thus require some local contral over the electric and magnetic field. This could be achieved using an externally applied global magnetic field that is modified locally to the desired value using a current-carrying wire (which could be used as an electrostatic gate at the same time). Although this requires more advanced sample fabrication, the pay-off in terms of a *T*_2_* time several orders of magnitude longer than for single electrons would be substantial. It is worth noting that a longer *T*_2_* time also makes protocols for dynamical decoupling (“spin echo”) more effective, which should lead to a longer *T*_2_ time as well^2^.

The four-electron singlet-triplet qubit design we propose can be useful in any coupled QD system where *in situ* control over the tunnel coupling is impossible. This includes not only self-assembled InGaAs CQDs as studied here, but also vertical CQDs inside etched pillars^19^ or semiconductor nanowires^29^. In contrast, the benefits of a four-electron system are not as clear for lateral CQDs defined electrostatically using gates on top of a two-dimensional electron gas. These systems, which have been used extensively in the context of transport experiments^2^,^10^,^11^, offer several ways to control the two-electron exchange splitting between *S* and *T*_0_, e.g. with a dedicated gate (which directly tunes the inter-dot tunneling barrier), or by applying a perpendicular magnetic field (which reduces the wavefunction overlap of the two dots).

## Methods

### Sample structure

The device, containing two layers of self-assembled InGaAs QDs separated by a 9 nm GaAs tunnel barrier and embedded in a GaAs Schottky diode, is grown by molecular beam epitaxy on a (100) GaAs substrate. QDs in the lower layer nucleate randomly, producing a strain field that facilitates nucleation of QDs in the upper layer, leading to pairs of vertically stacked QDs^30^,^31^. The emission wavelength of the QDs is blue-shifted into the near-infrared (~950-980 nm) by reducing the QD thickness in both layers using the partially-covered-island technique. To fill the CQD with four electrons, we apply an appropriate voltage *V* between the Si-doped *n*^+^-GaAs back contact (30 nm below the bottom QD layer) and a semi-transparent top gate (2 nm of Ti plus 6 nm of Au), deposited after growth. An AlGaAs layer of 20 nm thickness is incorporated 10 nm below the top surface to block current through the device.

### Measurement techniques

The device is mounted on a three-axis piezoelectric nano-positioning stack in a liquid-helium bath cryostat operating at 4.2 K. We use a single aspheric lens with a numerical aperture of 0.55 to focus the excitation laser to a near-diffraction limited spot on the sample, addressing a single CQD pair. To measure its photoluminescence, we use a confocal setup to excite the CQD non-resonantly with a 780 nm laser and collect the resulting luminescence through the same lens. The PL is analysed using a 75 cm grating spectrometer equipped with a liquid-nitrogen cooled charge-coupled device, which has a spectral resolution of ~30 μeV.

To perform high-resolution spectroscopy limited by the ~5 μeV CQD linewidth, we detect the differential transmission^24^ of a linearly polarized resonant laser, using a silicon photodiode placed directly below the sample. Alternatively, we collect the resonance fluorescence^25^,^26^ generated by the single CQD pair in focus, and detect it with the grating spectrometer described above. In this case, a cross-polarized detection scheme suppresses the excitation laser by six to seven orders of magnitude to prevent it from overwhelming the detector.

When comparing resonant measurements (such as Fig. 3a) with non-resonant ones (Fig. 2), a ~200 mV shift of all the resonances in gate voltage can be seen. This shift is due to the fact that the 780 nm laser used for PL produces charges in the GaAs and the wetting layer around the QD, leading to a partial screening of the applied gate voltage and a corresponding *V*-shift of all the resonances^32^.

## Author Contributions

K.M.W. and J.M.E. conducted the experiments, J.M.-S. grew the sample containing the InGaAs quantum dots and J.M.E. wrote the paper. All authors discussed the results, analysed the data and commented on the manuscript.

## Supplementary Material

Supplementary Information Supplementary material: Magnetically tunable singlet-triplet spin qubit in a four-electron InGaAs coupled quantum dot

## References

[b1] Warburton R. J. Single spins in self-assembled quantum dots. Nature Mat. 12, 483–493 (2013). 10.1038/nmat3585 23695745

[b2] Hanson R., Kouwenhoven L. P., Petta J. R., Tarucha S. & Vandersypen L. M. K. Spins in few-electron quantum dots. Rev. Mod. Phys. 79, 1217–1265 (2007).

[b3] Urbaszek B. *et al.* Nuclear spin physics in quantum dots: An optical investigation. Rev. Mod. Phys. 85, 79–133 (2013).

[b4] Chekhovich E. A. *et al.* Nuclear spin effects in semiconductor quantum dots. Nature Mat. 12, 494 (2013). 10.1038/nmat3652 23695746

[b5] Lidar D. A., Chuang I. L. & Whaley K. B. Decoherence-free subspaces for quantum computation. Phys. Rev. Lett. 81, 2594–2597 (1998).

[b6] Kim D., Carter S. G., Greilich A., Bracker A. S. & Gammon D. Ultrafast optical control of entanglement between two quantum-dot spins. Nature Phys. 7, 223–229 (2011).

[b7] Elzerman J. M., Weiss K. M., Miguel-Sanchez J. & Imamoglu A. Optical amplification using Raman transitions between spin-singlet and spin-triplet states of a pair of coupled In-GaAs quantum dots. Phys. Rev. Lett. 107, 017401 (2011). 21797571 10.1103/PhysRevLett.107.017401

[b8] Weiss K. M., Elzerman J. M., Delley Y. L., Miguel-Sanchez J. & Imamoglu A. Coherent two-electron spin qubits in an optically active pair of coupled InGaAs quantum dots. Phys. Rev. Lett. 109, 107401 (2012). 23005324 10.1103/PhysRevLett.109.107401

[b9] Kuhlmann A. V. *et al.* Charge noise and spin noise in a semiconductor quantum device. Nature Phys. 9, 570 (2013).

[b10] Dial O. E. *et al.* Charge noise spectroscopy using coherent exchange oscillations in a singlet-triplet qubit. Phys. Rev. Lett. 110, 146804 (2013). 10.1103/PhysRevLett.110.146804 25167023

[b11] Kloeffel C. & Loss D. Prospects for spin-based quantum computing. Annu. Rev. Condens. Matter Phys. 4, 51 (2013).

[b12] Imamoglu A. *et al.* Quantum information processing using quantum dot spins and cavity QED. Phys. Rev. Lett. 83, 4204 (1999).

[b13] Trifunovic L., Pedrocchi F. L. & Loss D. Long-range interaction of singlet-triplet qubits via ferromagnets. arXiv:1305.2451. (2013).

[b14] Press D., Ladd T. D., Zhang B. & Yamamoto Y. Complete quantum control of a single quantum dot spin using ultrafast optical pulses. Nature 456, 218–221 (2008). 19005550 10.1038/nature07530

[b15] Kim E. D. *et al.* Fast spin rotations by optically controlled geometric phases in a charge-tunable InAs quantum dot. Phys. Rev. Lett. 104, 167401 (2010). 20482081 10.1103/PhysRevLett.104.167401

[b16] Greilich A., Carter S. G., Kim D., Bracker A. S. & Gammon D. Optical control of one and two hole spins in interacting quantum dots. Nature Photon. 5, 703–709 (2011).

[b17] De Greve K. *et al.* Ultrafast coherent control and suppressed nuclear feedback of a single quantum dot hole qubit. Nature Phys. 7, 872–878 (2011).

[b18] Godden T. M. *et al.* Coherent optical control of the spin of a single hole in an InAs/GaAs quantum dot. Phys. Rev. Lett. 108, 017402 (2012). 22304289 10.1103/PhysRevLett.108.017402

[b19] Kouwenhoven L. P., Austing D. G. & Tarucha S. Few-electron quantum dots. Rep. Prog. Phys. 64, 701–736 (2001).

[b20] Karrai K. *et al.* Hybridization of electronic states in quantum dots through photon emission. Nature 427, 135 (2004). 14712271 10.1038/nature02109

[b21] Stinaff E. A. *et al.* Optical signatures of coupled quantum dots. Science 311, 636–639 (2006). 16410487 10.1126/science.1121189

[b22] Krenner H. J. *et al.* Direct observation of controlled coupling in an individual quantum dot molecule. Phys. Rev. Lett. 94, 057402 (2005). 15783693 10.1103/PhysRevLett.94.057402

[b23] Seidl S. *et al.* Resonant transmission spectroscopy on the p to p transitions of a charge tunable InGaAs quantum dot. Appl. Phys. Lett. 92, 153103 (2008).

[b24] Karrai K. & Warburton R. J. Optical transmission and reflection spectroscopy of single quantum dots. Superlattices and Microstructures 33, 311–337 (2003).

[b25] Muller A. *et al.* Resonance fluorescence from a coherently driven semiconductor quantum dot in a cavity. Phys. Rev. Lett. 99, 187402 (2007). 17995437 10.1103/PhysRevLett.99.187402

[b26] Vamivakas A. N., Zhao Y., Lu C. Y. & Atatüre M. Spin-resolved quantum-dot resonance fluorescence. Nature Phys. 5, 198 (2009). 10.1038/nature09359 20844531

[b27] Atature M. *et al.* Quantum-dot spin-state preparation with near-unity fidelity. Science 312, 551–553 (2006). 16601152 10.1126/science.1126074

[b28] Xu X. *et al.* Fast spin state initialization in a singly charged InAs-GaAs quantum dot by optical cooling. Phys. Rev. Lett. 99, 097401 (2007). 17931035 10.1103/PhysRevLett.99.097401

[b29] Skold N. *et al.* Microphotoluminescence studies of tunable wurtzite InAs0.85P0.15 quantum dots embedded in wurtzite InP nanowires. Phys. Rev. B 80, 041312 (2009).

[b30] Xie Q., Madhukar A., Chen P. & Kobayashi N. P. Vertically self-organized InAs quantum box islands on GaAs(100). Phys. Rev. Lett. 75, 2542 (1995). 10059338 10.1103/PhysRevLett.75.2542

[b31] Kiravittaya S., Rastelli A. & Schmidt O. G. Advanced quantum dot configurations. Rep. Prog. Phys. 72, 046502 (2009).

[b32] Houel J. *et al.* Probing single-charge fluctuations at a GaAs/AlAs interface using laser spectroscopy on a nearby InGaAs quantum dot. Phys. Rev. Lett. 108, 107401 (2012). 22463453 10.1103/PhysRevLett.108.107401

[b33] Fält S. *et al.* Strong electron-hole exchange in coherently coupled quantum dots. Phys. Rev. Lett. 100, 106401 (2008). 18352214 10.1103/PhysRevLett.100.106401

