# Supplementary material for: Magnetically tunable singlet-triplet spin qubit in a four-electron InGaAs coupled quantum dot
Source: Sci Rep. 2013 Nov 1;3:3121. doi: 10.1038/srep03121 (PMC3814830; doi:10.1038/srep03121)
Supplement: Supplementary Information — Supplementary material: Magnetically tunable singlet-triplet spin qubit in a four-electron InGaAs coupled quantum dot [file srep03121-s1.pdf]

Supplementary material:
Magnetically tunable singlet-triplet spin qubit in a four-electron InGaAs coupled quantum dot

K. M. Weiss[1], J. Miguel-Sanchez[1], and J. M. Elzerman[1,2,*]
[1] *Institute of Quantum Electronics, ETH Zurich, CH-8093 Zurich, Switzerland*
[2] *London Centre for Nanotechnology and Department of Electronic & Electrical Engineering, University College London, London WC1H 0AH, UK*
[*] *j.elzerman@ucl.ac.uk*

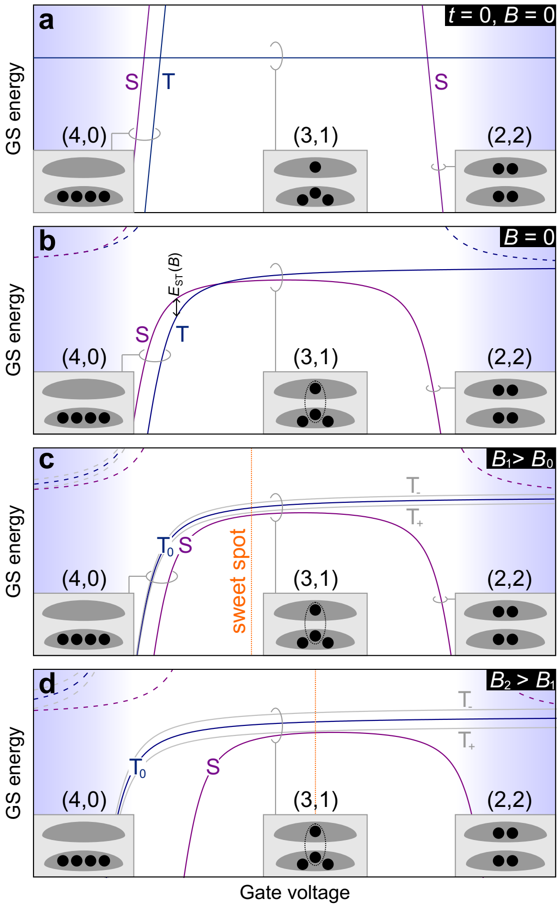

Figure 1: Schematic energy diagrams of the four-electron states versus gate voltage. (a) Ground-state energies in the absence of tunnel coupling and magnetic field. For very negative voltage V, the $(4,0)$ charge configuration (with all four electrons in the lower QD) is the ground state. The three degenerate $(4,0)$ triplet (T) states have lower energy than the $(4,0)$ singlet (S) due to the exchange energy. As V is increased, the $(3,1)$ charge configuration becomes the ground state at some value of V. Due to the absence of tunnel coupling, the different charge states simply cross and therefore the $(3,1)S$ and T states are degenerate throughout the gate-voltage range. Making V even more positive, at some value of V the $(2,2)$ charge configuration becomes the ground state. This state corresponds to two electrons occupying the lowest s-orbital and forming a spin singlet in each QD. The $(2,2)$ triplet states are $\sim 20\,\text{meV}$ higher in energy, since they involve a p-orbital in one of the two dots. Therefore, they are not visible in this figure, as they would appear to the right of the range shown here. (b) Ground-state energies in the presence of tunnel coupling but without magnetic field. All crossings between states with the same spin character have become anti-crossings, leading to an exchange splitting E_{ST} between the $(3,1)$ spin singlet and triplet states. The anti-crossing between the $(3,1)T$ and $(2,2)T$ states is not visible, as it would appear to the right of the range shown here. No sweet spot with $dE_{ST}/dV = 0$ occurs anywhere in the gate-voltage range. (c) When the magnetic field is increased above B_0, so that the energy of $(4,0)S$ becomes lower than that of $(4,0)T_0$, a sweet spot appears for a particular gate voltage (indicated by the orange dashed line). (d) When B is increased even further, the sweet spot moves to the right, and E_{ST} at the sweet spot increases.

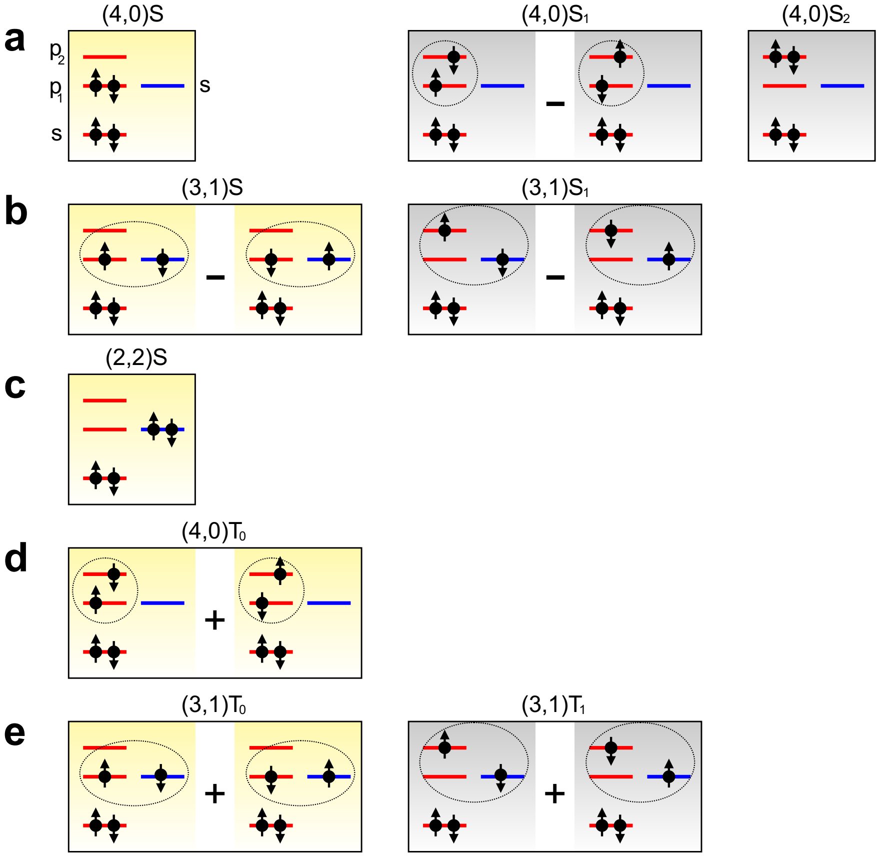

Figure 2: Schematic diagrams representing the various four-electron states that make up the qubit states. The three red lines indicate the s, p1 and p2 orbital in QD-R, whereas the blue line indicates the s-orbital in QD-B. (a-c) Six singlet states that make up the qubit state S. (d-e) Three triplet states that make up the qubit state T_0. The (2,2) triplet states are left out of the model, as they would involve a p-orbital in QD-B. The relative weights of each configuration in S and T_0 depend on the value of the gate voltage V. When V is relatively small, the (4,0) states dominate; making V larger first increases the weigth of (3,1) and then also (2,2). For the gate voltages in our experiment, the (4,0) and (3,1) contributions are largest, and (2,2) can be neglected. Yellow diagrams represent the lowest-energy configurations, whereas grey diagrams represent higher-lying states. Thus, for each gate voltage the yellow configurations dominate in both S and T_0. Normalization constants are omitted from the diagrams for clarity.

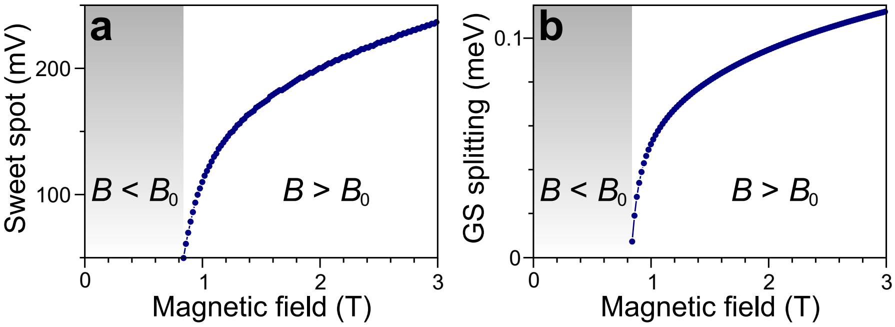

Figure 3: Simulated position of the sweet spot and the corresponding singlet-triplet splitting versus magnetic field. The main parameters in the simulation are: the tunnelling matrix element ($t = 0.75\,\mathrm{meV}$), the on-site Coulomb energy in QD-R and QD-B ($V_R = V_B = 25\,\mathrm{meV}$), the inter-dot Coulomb energy ($V_{BR} = 15\,\mathrm{meV}$), the exchange energy ($K = 1.5\,\mathrm{meV}$), the zero-field splitting of the p-states in QD-R ($\delta\epsilon_p(B = 0) = 0.5\,\mathrm{meV}$), and the splitting of the p-states with B ($\approx 1.8\,\mathrm{meV/T}$). (a) Gate voltage where the sweet spot occurs versus B. (Note that the absolute value of the sweet spot voltage is not relevant, only the shift is meaningful here.) As explained in supplementary Fig. S1, there is no sweet spot for $B < B_0$ (which is ~ 0.8 T for the parameters used in this simulation). The sweet spot appears above B_0, and it first moves very strongly with B before the shift becomes approximately linear. In practice, the $(3, 1)$ regime extends across a finite region in V, and is cut off by the $(4, 1)$ and $(3, 0)$ charge regimes. This limits the achievable shift of the sweet spot. (b) Energy splitting between S and T_0 at the sweet spot. The splitting E_{ST} can be tuned over a range of ~ 0.1 meV for the parameters chosen here. However, this maximum range is limited in practice by the finite extent of the $(3, 1)$ charge regime.
